# Cryotolerance of Stallion Spermatozoa Relies on Aquaglyceroporins rather than Orthodox Aquaporins

**DOI:** 10.3390/biology8040085

**Published:** 2019-11-12

**Authors:** Ariadna Delgado-Bermúdez, Federico Noto, Sebastián Bonilla-Correal, Estela Garcia-Bonavila, Jaime Catalán, Marion Papas, Sergi Bonet, Jordi Miró, Marc Yeste

**Affiliations:** 1 Biotechnology of Animal and Human Reproduction (TechnoSperm), Unit of Cell Biology, Department of Biology, Institute of Food and Agricultural Technology, Faculty of Sciences, University of Girona, 17003 Girona, Spain; ariadna.delgado@udg.edu (A.D.-B.); fnoto83@gmail.com (F.N.); estela.garcia@udg.edu (E.G.-B.); sergi.bonet@udg.edu (S.B.); 2 Equine Reproduction Service, Department of Animal Medicine and Surgery, Faculty of Veterinary Sciences, Autonomous University of Barcelona, 08193 Bellaterra (Cerdanyola del Vallès), Spain; sebastian.bonilla@e-campus.uab.cat (S.B.-C.); dr.jcatalan@gmail.com (J.C.); papas.marion@gmail.com (M.P.); jordi.miro@uab.cat (J.M.)

**Keywords:** acetazolamide, aquaporins, phloretin, propanediol, sperm, stallion

## Abstract

Aquaporins (AQPs), a family of ubiquitous water channels divided into orthodox AQPs, aquaglyceroporins (GLPs), and superAQPs, are present in stallion spermatozoa. The aim of this study was to elucidate the functional relevance of each group of AQPs during stallion sperm cryopreservation through the use of three different inhibitors: acetazolamide (AC), phloretin (PHL) and propanediol (PDO). Sperm quality and function parameters were evaluated in the presence or absence of each inhibitor in fresh and frozen–thawed samples. In the presence of AC, different parameters were altered (*p* < 0.05), but not in a concentration- or time-depending manner. PHL was found to decrease sperm motility, viability, acrosome integrity, and the percentages of spermatozoa with low membrane lipid disorder, high mitochondrial membrane potential (MMP) and high intracellular levels of calcium and superoxides (*p* < 0.05). Finally, the sperm motility, viability, acrosome integrity, the percentages of spermatozoa with low membrane lipid disorder, high MMP and high intracellular calcium levels were higher (*p* < 0.05) in PDO treatments than in the control. The sperm response to AC, PHL and PDO indicates that GLPs, rather than orthodox AQPs, play a crucial role during stallion sperm cryopreservation. Furthermore, post-thaw sperm quality was higher in PDO treatments than in the control, suggesting that this molecule is a potential permeable cryoprotectant.

## 1. Introduction

The permeability of the plasma membrane to water and solutes is crucial for proper cell function and homeostasis. Because of the amphipathic nature of the plasma membrane, the simple diffusion of water molecules does not occur at high rates [1]. Aquaporins (AQPs) are a family of ubiquitous transmembrane proteins that allow for the facilitated diffusion of water. Some AQPs are also permeable to small solutes [2]. To date, 13 AQPs (AQP0–AQP12) have been identified in mammalian cells and have been classified into three different groups (orthodox AQPs, aquaglyceroporins (GLPs), and superAQPs), which differ in their sequence and solute permeability. The orthodox AQP group includes AQP0, AQP1, AQP2, AQP4, AQP5, AQP6, and AQP8, which are exclusively permeable to water. The group of GLPs that is permeable to water, glycerol, urea and other small electrolytes comprises AQP3, AQP7, AQP9 and AQP10. Finally, AQP11 and AQP12 are members of the superAQPs group, which are localized in the membranes of intracellular organelles and are involved in the transport of water and glycerol. Even if ubiquitous, the presence of AQPs varies between cell types and species. In mammalian spermatozoa, AQP1 has been identified in porcine [3]; AQP3, AQP7 and AQP11 in equine [4], porcine [5,6], murine [7,8,9], human [10,11] and bovine species [12,13]; AQP8 has been found in mouse [8] and human spermatozoa [10,11]; and AQP9 is present in boar spermatozoa [14]. In the male gamete, AQPs are involved in the regulation of cell volume and osmotic balance, which are crucial for spermatogenesis [15] and post-ejaculation events, including the activation of sperm motility upon ejaculation and sperm adaptation to the female environment (reviewed in [2]).

Cryopreservation, which is the most efficient method for the long-term storage of stallion spermatozoa, causes a drastic impairment in sperm quality at both lethal and sub-lethal temperature levels. The hyperosmotic shock during freezing and the following hypotonic stress during thawing induce a dramatic modification of the sperm cell volume that injuries the cytoskeleton, the mitochondria and the plasma membrane (reviewed in [16]). Mitochondria are the most sensitive organelle to osmotic stress and, when damaged, these organelles are a major source of oxidative stress through the generation of reactive oxygen species (ROS) [17]. Moreover, alterations in the plasma membrane affect embedded and membrane-associated proteins. This, in turn, affects specific signaling pathways and may detrimentally impact sperm fertilizing ability [18].

It is worth mentioning that there is a high variability between and within stallions in the ability of their ejaculates to withstand cryopreservation, i.e., cryotolerance or freezability. This inter- and intra-individual variability has also been described in other mammalian species [19,20,21]. Good (GFE) and poor freezability ejaculates (PFE) differ in their post-thaw sperm quality and function parameters, such as sperm membrane integrity, motility [22], ROS production and mitochondrial membrane potential (MMP) [23], all of which may ultimately affect the fertilizing capacity [24]. 

During the last decade, the efforts to improve the efficiency of sperm cryopreservation protocols have been focused on the use of alternative cryoprotectant agents (CPAs) and antioxidants to reduce osmotic and oxidative stresses, respectively (reviewed in [16]). In addition, the presence of damaged and non-viable sperm cells after thawing, which releases factors that have deleterious effects on viable spermatozoa, has evidenced the importance of selecting stallion spermatozoa after cryopreservation [25]. In spite of these advances, increasing cryopreservation efficiency in stallion spermatozoa still needs further optimization through the prediction of sperm cryotolerance (i.e., identification of GFE). In this context, AQPs are potential freezability biomarkers, since their permeability to water and small molecules are crucial for sperm’s response to osmotic stress. In fact, the AQP involvement in sperm cryopreservation has previously been confirmed in bull [12,13], boar [26,27] and stallion spermatozoa [4]. In effect, AQP3 and AQP7 are related to the cryotolerance of boar spermatozoa [28], AQP7 [5] and AQP11 [13] are associated with that of bull spermatozoa, and AQP3, AQP7, and AQP11 are related to stallion sperm freezability [4]. As one may assume that the inhibition of these AQPs may affect the sperm’s ability to withstand cryopreservation, the present study aimed to elucidate the functional relevance of orthodox AQPs and GLPs during stallion sperm cryopreservation by using three separate inhibitors (phloretin (AC), phloretin (PHL) and propanediol (PDO)). The sperm response to AQP-inhibition indicates that GLPs, rather than orthodox AQPs, play a crucial role during stallion sperm cryopreservation. Moreover, post-thaw sperm quality is higher in the presence of PDO than in the control, suggesting that this molecule could be added to cryopreservation media as a permeable cryoprotectant.

## 2. Materials and Methods 

### 2.1. Stallions and Ejaculates

A total of 12 ejaculates coming from different stallions (n = 12) were used. Animals were housed at the Equine Reproduction Service, Autonomous University of Barcelona (Spain), which is a European Union (EU)-approved equine semen collection center (Authorization code: ES09RS01E) that operates under strict protocols of animal welfare and health control. Since all stallions used in this study were semen donors and were housed at the Equine Reproduction Service, the local ethics committee at our University indicated that no further ethics authorization was required. 

Semen samples were collected using a Hannover-type artificial vagina (Minitüb GmbH, Tiefenbach, Germany) with an in-line nylon mesh filter to separate the gel fraction. Gel-free semen ere subsequently diluted 1:5 (v:v) in a Kenney extender [29], previously warmed at 37 °C. Sperm concentration was assessed with a Neubauer chamber (Paul Marienfeld GmbH & Co. KG, Lauda-Köningshofen, Germany), and each sample was split into two different fractions. The first one was used to assess the quality of the fresh semen, whereas the other was divided into nine different sub-fractions that were cryopreserved in the presence or absence of different concentrations of the three AQP-inhibitors.

### 2.2. AQP Inhibitors

Prior to cryopreservation, three AQP inhibitors were added to semen samples: 1,3-propanediol (PDO, Sigma-Aldrich, St. Louis, MO, USA), acetazolamide (AC, Sigma-Aldrich), and phloretin (PHL, Sigma-Aldrich). PDO was diluted in the commercial freezing extender used for cryopreservation (see next section) to a working concentration of 100 mmol/L; AC was diluted in dimethyl sulfoxide (DMSO, Sigma-Aldrich) to a working concentration of 450 mmol/L, and PHL was diluted in methanol (Fisher Chemical, ThermoFisher Scientific; Waltham, MA, USA) to a working concentration of 365 mmol/L. Each inhibitor was assayed at the following concentrations, which were chosen according to preliminary experiments conducted in our laboratory and previous studies [27]: 0.1, 1 and 10 mmol/L for PDO; 250, 500 and 1000 µmol/L for AC; and 350 and 800 µmol/L for PHL. It is worth mentioning that, in the case of treatments containing PDO and AC, samples were exposed to methanol or DMSO at concentrations lower than 0.5% (v/v). These concentrations showed no detrimental effects on sperm quality parameters (data not shown).

### 2.3. Stallion Sperm Cryopreservation

The cryopreservation of stallion spermatozoa was performed in order to assess how the inhibition of AQPs from different groups affected sperm cryotolerance. The fraction of the ejaculate intended for cryopreservation was centrifuged in a programmable centrifuge (Medifriger BL-S; JP Selecta S.A., Barcelona, Spain) at 600× *g* and 20 °C for 15 min, and the supernatants were discarded. Pellets were subsequently resuspended in 2 mL of a BotuCRYO™ commercial extender (Botupharma, Botucatu, Brazil), and sperm concentration, motility and membrane integrity were evaluated for the subsequent adjustment of sperm concentration to 2 × 10^6^ viable spermatozoa per mL. In this step, eight aliquots of 1 mL of semen each were added with AQP inhibitors (see Section 2.2), and the remaining semen was used as a control. Treatments were as follows: AC at 250 µmol/L (AC250); AC at 500 µmol/L (AC500); AC at 1000 µmol/L (AC1000); PDO at 0.1 mmol/L (PDO0.1); PDO at 1 mmol/L (PDO1); PDO at 10 mmol/L (PDO10); PHL at 350 µmol/L (PHL350); and PHL at 800 µmol/L (PHL800). Samples were subsequently packaged into 0.5 mL straws (Minitüb) and frozen in a controlled-rate freezer (Ice-Cube 14S-B; Minitüb), using the following cooling rates: i) −0.25 °C/min from 20 to 5 °C (60 min), ii) −4.75 °C/min from 5 to −90 °C (20 min), and iii) −11.11 °C/min from −90 to −120 °C (2.7 min). Finally, straws were plunged into liquid nitrogen (−196 °C) for storage.

Frozen–thawed sperm quality was evaluated after thawing. Two straws per ejaculate were thawed at 37 °C by immersion in a water bath for 20 s. The content of these straws was then diluted 1:3 (v:v) in a pre-warmed Kenney medium. After that, samples were incubated at 37 °C for 2 h, and sperm quality was assessed twice: at 10 min (0 h) and 2 h post-thaw.

### 2.4. Sperm Motility

Sperm motility was evaluated before and after freeze–thawing through a computer-assisted sperm analysis (CASA) system that consisted of a phase-contrast microscope (Olympus BX41; Olympus, Tokyo, Japan) equipped with a video camera and ISAS software (Integrated Sperm Analysis System V1.0; Proiser SL, Valencia, Spain). The assessment of sperm motility in extended samples was performed after 15 min of incubation at 37 °C; frozen–thawed samples were evaluated after 10 min (0 h) and 2 h of thawing. Three replicates of 1000 spermatozoa per sample and time points were evaluated using a pre-warmed (at 37 °C) Makler counting chamber (Sefi-Medical Instruments, Haifa, Israel) and observed under a negative phase-contrast field (Olympus 10 × 0.30 PLAN objective, Olympus). 

For each motility assessment, the evaluation of the following parameters was performed: total motility (TMOT, %), progressive sperm motility (PMOT, %); curvilinear velocity (VCL, µm·s^−1^); straight line velocity (VSL, µm·s^−1^); average path velocity (VAP, µm·s^−1^); amplitude of lateral head displacement (ALH, µm); beat cross frequency (BCF, Hz); linearity (LIN, %), which was calculated assuming that LIN = VSL/VCL × 100; straightness (STR, %), resulting from VSL/VAP × 100; and motility parameter wobble (WOB, %), obtained from VAP/VCL × 100. A sperm cell was considered to be motile when its VAP was higher than 10 µm/s, and it was considered to be progressively motile when its STR was higher than 75%. For each parameter, the corresponding mean ± standard error of the mean (SEM) was calculated.

### 2.5. Flow Cytometry Analyses

Flow cytometry analyses were performed in order to evaluate different sperm quality parameters in both fresh and frozen–thawed sperm samples: viability, acrosome integrity, membrane lipid disorder, MMP, intracellular calcium levels, and intracellular levels of superoxide (O_2_^●−^) and peroxides (H_2_O_2_). Samples were diluted to a final concentration of 1 × 10^6^ sperm/mL with 4-(2-hydroxyethyl)-1-piperazineethanesulfonic acid (HEPES) buffered saline solution (10 mmol/L HEPES, 150 mmol/L NaCl, 10% BSA; pH = 7.4) prior to staining with the corresponding fluorochromes (ThermoFisher Scientific, Waltham, MA, USA). After that, samples were incubated at 38 °C in the dark, and a total of three replicates per sample and parameter were evaluated.

Flow cytometry analyses were performed using a Cell Laboratory QuantaSC^TM^ cytometer (Beckman Coulter; Fullerton, CA, USA), and samples were excited with an argon ion laser (488 nm) set at a power of 22 mW. Cell diameter/volume was determined using the Cell Lab Quanta™ SC cytometer through the Coulter principle for volume assessment, which is based on the changes in electrical resistance produced in an electrolyte solution by suspended, non-conductive particles. In this system, forward scatter (FS) is replaced by electronic volume (EV). The EV-channel was calibrated using 10 µm flow-check fluorospheres (Beckman Coulter), and this size of beads was positioned at channel 200 on the EV scale.

Three different optical filters were used: FL1 (Dichroic/Splitter, DRLP (dichroic long pass): 550 nm, BP (band pass) filter: 525 nm, detection width: 505–545 nm), FL2 (DRLP: 600 nm, BP filter: 575 nm, detection width: 560–590 nm) and FL3 (LP (long pass) filter: 670 nm/730 nm, detection width: 655–685 nm). While FL1 allowed for the detection of green fluorescence from SYBR14, *Arachis hypogaea* lectin—peanut agglutinin—conjugated with fluorescein isothiocyanate (PNA-FITC), YO-PRO-1, JC1 (5,5’,6,6’-tetrachloro-1,1’,3,3’tetraethyl-benzimidazolylcarbocyanine iodide) monomers (JC1_mon_), Fluo3 and 2’,7’-dichlorofluorescein (DCF); FL2 was used to detect orange fluorescence from JC1 aggregates (JC1_agg_); and FL3 allowed for the detection of red fluorescence from propidium iodide (PI), merocyanine 540 (M540), Rhod5 and ethidium (E). Signals were logarithmically amplified, and the adjustment of photomultiplier settings was performed according to particular staining methods. 

The sheath flow rate was set at 4.17 µL/min, and EV and side scatter (SS) were measured and linearly recorded for all particles. The analyzer threshold on the EV channel was adjusted to exclude subcellular debris (particle diameter <7 µm) and cell aggregates (particle diameter >12 µm), and the sperm-specific events were positively gated on the basis of EV/SS distributions.

Data obtained from flow cytometry evaluations were analyzed with Flowing Software (Ver. 2.5.1; University of Turku, Turku, Finland), and the recommendations of the International Society for Advancement of Cytometry (ISAC) were adhered. Following Petrunkina et al. [30], the events corresponding to the double negative-stained particles of all protocols except SYBR14/PI were corrected using the percentage of non-sperm debris particles of the SYBR14^−^/PI^−^ population. The percentages of all the other sperm populations were recalculated. Finally, the corresponding mean ± SEM was calculated for each parameter.

#### 2.5.1. Plasma Membrane and Acrosome Integrity

Plasma membrane and acrosome integrity were evaluated through two different tests: SYBR14/PI and PNA-FITC/PI.

On the one hand, the LIVE/DEAD sperm viability kit (Molecular Probes, Eugene, OR, USA) was used following the protocol of Garner and Johnson [31]. In brief, sperm were incubated with SYBR14 at a final concentration of 100 nmol/L for 10 min, and PI was added at a final concentration of 12 µmol/L prior to an additional incubation of 5 min. Three different sperm populations were observed: 1) viable, green-stained spermatozoa (SYBR14^+^/PI^−^); 2) non-viable, red-stained spermatozoa (SYBR14^−^/PI^+^); and 3) non-viable spermatozoa stained in both green and red (SYBR14^+^/PI^+^). The remaining population in the dot-plots, which corresponded to unstained, non-sperm particles (SYBR14^−^/PI^−^), was not included in the calculation of the final percentages of each sperm population (SYBR14^+^/PI^−^, SYBR14^−^/PI^+^, and SYBR14^+^/PI^+^). The SYBR14 fluorescence spill over into the FL3 channel was compensated for (2.45%).

In the second case, spermatozoa were co-stained with PNA-FITC and PI, following the procedure described by Nagy et al. [32] with minor modifications. Briefly, spermatozoa were incubated with PNA-FITC (final concentration: 2.5 µg/mL) and with PI (12 µmol/L) for 10 min. Flow cytometry dot-plots allowed for the identification of four different populations: 1) spermatozoa with an intact plasma membrane (PNA-FITC^−^/PI^−^); 2) spermatozoa with a damaged plasma membrane (PNA-FITC^+^/PI^−^); 3) spermatozoa with a damaged plasma membrane and a partially altered outer acrosome membrane (PNA-FITC^+^/PI^+^); and 4) spermatozoa with a damaged plasma membrane and a lost outer acrosome membrane (PNA-FITC^−^/PI^+^). Thereafter, spermatozoa were classified into two different categories: (a) spermatozoa with an intact plasma membrane and acrosome (Population 1; PNA-FITC^−^/PI^−^); and (b) spermatozoa with acrosome and/or plasma membrane damage, which included Populations 2, 3 and 4. Events appearing in the PNA-FITC^−^/PI^−^ quadrant were corrected using the percentages of non-sperm debris particles found in the SYBR14^−^/PI^−^ quadrant; the percentages of the other three populations were recalculated. PNA-FITC fluorescence spill over into the FL3 channel was compensated for (2.45%).

#### 2.5.2. Sperm Membrane Lipid Disorder

The evaluation of sperm membrane lipid disorder was performed according the protocol from Rathi et al. [33] with minor modifications [34], using M540 and YO-PRO-1. M540 detected the decrease in packing order of phospholipids in the outer monolayer of the plasma membrane. Samples were incubated with M540 (final concentration: 2.6 µmol/L) and YO-PRO-1 (final concentration: 25 nmol/L) for 10 min. Four populations were observed in flow cytometry dot-plots: 1) non-viable spermatozoa with low membrane lipid disorder (M540^−^/YO-PRO-1^+^); 2) non-viable spermatozoa with high membrane lipid disorder (M540^+^/YO-PRO-1^+^); 3) viable spermatozoa with low membrane lipid disorder (M540^−^/YO-PRO-1^−^); and 4) viable spermatozoa with high membrane lipid disorder (M540^+^/YO-PRO-1^−^). The percentages of viable spermatozoa with low membrane lipid disorder (M540^−^/YO-PRO-1^−^) were corrected using proportions of non-sperm debris particles found in the SYBR-14^−^/PI^−^ quadrant; the percentages of the other three populations were recalculated. Data were not compensated for.

#### 2.5.3. Mitochondrial Membrane Potential (MMP)

JC1-staining was used for the assessment of MMP, following the protocol of Ortega-Ferrusola et al. [35] with minor modifications. In brief, samples were incubated in the presence of JC1 at a final concentration of 0.3 µmol/L for 30 min. JC1 molecules form aggregates (JC1_agg_) in the presence of high MMP, whereas JC1 molecules remain monomers in the presence of low MMP (JC1_mon_). Three different populations were identified in flow cytometry dot-plots: 1) spermatozoa with low MMP (JC1_mon_; FL1^+^/FL2^−^); 2) spermatozoa with high MMP (JC1_agg_; FL1^−^/FL2^+^; and 3) spermatozoa with heterogeneous mitochondria (JC1_agg_ and JC1_mon_; FL1^+^/FL2^+^) in the same cell. The percentages of double-negative particles (FL1^−^/FL2^−^) were corrected using the proportions of SYBR14^−^/PI^−^; the percentages of the other populations were recalculated. Spermatozoa with high MMP resulted from the sum of Populations 2 and 3. FL1 spill-over into the FL2 channel was compensated for (70.29%).

#### 2.5.4. Intracellular Calcium Levels

For the evaluation of intracellular calcium levels, two different co-staining tests were performed: Fluo3-AM (acetoxymethyl, ester form)/PI and Rhod5-N/YO-PRO-1.

In the first test, intracellular calcium levels were evaluated with Fluo3-AM, which penetrates cell membranes and has more affinity for the calcium residing in the sperm mid-piece [36]. This test was performed according to the protocol of Harrison et al. [37] that was modified by Kadirvel et al. [38]. In brief, spermatozoa were incubated in the presence of 1 µmol/L of Fluo3-AM and 12 µmol/L of PI for 10 min. Four different sperm populations were identified in dot-plots: 1) non-viable spermatozoa with low intracellular calcium levels (Fluo3^−^/PI^+^); 2) non-viable spermatozoa with high intracellular calcium levels (Fluo3^+^/PI^+^); 3) viable spermatozoa with low intracellular calcium levels (Fluo3^−^/PI^−^); and 4) viable spermatozoa with high intracellular calcium levels (Fluo3^+^/PI^−^). The percentages of viable spermatozoa with low intracellular calcium levels (Fluo3^−^/PI^−^) were corrected using proportions of non-sperm debris particles found in the SYBR14^−^/PI^−^ quadrant; the percentages of the other three populations were recalculated. The spillover of PI into the FL1-channel and Fluo3 spill over into the FL3-channel were compensated for (28.72% and 2.45%, respectively).

Intracellular calcium levels were also assessed through Rhod-5N staining following the protocol described by Yeste et al. [36], in which Rhod-5N was found to have more affinity for the calcium residing in the sperm head. Briefly, samples were incubated with Rhod-5N at a final concentration of 5 µmol/L and YO-PRO-1 at a final concentration of 25 nmol/L for 10 min. Four different populations were identified in flow cytometry dot-plots: 1) non-viable spermatozoa with low levels of intracellular calcium (Rhod5^−^/YO-PRO-1^+^); 2) non-viable spermatozoa with high levels of intracellular calcium (Rhod5^+^/YO-PRO-1^+^); 3) viable spermatozoa with low levels of intracellular calcium (Rhod5^−^/YO-PRO-1^−^); and 4) viable spermatozoa with high levels of intracellular calcium (Rhod5^+^/YO-PRO-1^−^). The percentages of viable spermatozoa with low intracellular calcium levels (Rhod5N^−^/YO-PRO-1^−^) were corrected using the percentages of non-sperm debris particles found in the SYBR14^−^/PI^−^ quadrant; the percentages of the other three populations were recalculated. Rhod5 spill over into the FL1-channel was compensated for (3.16%).

#### 2.5.5. Intracellular Superoxide Levels (O_2_^−^)

The evaluation of intracellular superoxide (O_2_^−^) radical levels was performed following a modification of the protocol from Guthrie and Welch [39] through hydroethidine (HE) and YO-PRO-1 co-staining. HE is able to penetrate the sperm plasma membrane, and it is oxidized by O_2_^−^ to ethidium (E^+^) and other products at the intracellular environment. In brief, samples were incubated with HE (final concentration: 4 µmol/L) and YO-PRO-1 (final concentration: 40 nmol/L) for 20 min. Four different sperm populations were identified in flow cytometry dot-plots: 1) non-viable spermatozoa with low superoxide levels (E^−^/YO-PRO-1^+^); 2) non-viable spermatozoa with high superoxide levels (E^+^/YO-PRO-1^+^); 3) viable spermatozoa with low superoxide levels (E^−^/YO-PRO-1^−^); and 4) viable spermatozoa with high superoxide levels (E^+^/YO-PRO-1^−^). The percentages of viable spermatozoa with low superoxide levels (E^−^/YO-PRO-1^−^) were corrected using the percentages of non-sperm debris particles found in the SYBR14^−^/PI^−^ quadrant; the percentages of the other three populations were recalculated. YO-PRO-1 spill over into the FL3-channel was compensated for (5.06%).

#### 2.5.6. Intracellular Hydrogen Peroxide Levels (H_2_O_2_)

The determination of the intracellular levels of hydrogen peroxide (H_2_O_2_) through co-staining with 2’,7’-dichlorodihydrofluorescein diacetate (H_2_DCFDA) and PI fluorochromes was performed following the protocol from Guthrie and Welch [39] with minor modifications. H_2_DCFDA is a non-fluorescent probe that penetrates the sperm plasma membrane and is intracellularly de-esterified and converted into highly fluorescent, 2’,7’-dichlorofluorescein (DCF^+^) upon oxidation. In brief, samples were incubated with H_2_DCFDA (final concentration: 200 µmol/L) and PI (final concentration: 12 µmol/L) for 30 min. Four different populations were identified in flow cytometry dot-plots: 1) viable spermatozoa with high peroxide levels (DCF^+^/PI^−^); 2) non-viable spermatozoa with high peroxide levels (DCF^+^/PI^+^); 3) viable spermatozoa with low peroxide levels (DCF^−^/PI^−^); and 4) non-viable spermatozoa with low peroxide levels (DCF^−^/PI^+^). The percentages of viable spermatozoa with low peroxide levels (DCF^−^/PI^−^) were corrected using the percentages of non-sperm debris particles found in the SYBR14^−^/PI^−^ quadrant; the percentages of the other three populations were recalculated. DCF-spill over into the FL3-channel was compensated for (2.45%).

### 2.6. Statistical Analyses

All data were analyzed using a statistical package (IBM SPSS Statistics 25.0; Armonk, New York, NY, USA). Data were first tested for normal distribution (Shapiro–Wilk test) and homogeneity of variances (Levene test). Following this, the effects of each inhibitor and cryopreservation step (i.e. fresh, frozen–thawed at 0 h, and frozen–thawed at 2 h) were tested through a mixed model followed by the post-hoc Sidak test for pair-wise comparisons. The intra-subjects factor (i.e., repeated measures) was the cryopreservation step (i.e., fresh, frozen–thawed at 0 h, frozen–thawed at 2 h), and the inter-subjects factor was the treatment (C, and different concentrations of PDO, AC or PHL). The level of significance was set at *p* ≤ 0.05, and data are shown as mean ± SEM.

## 3. Results

As previously mentioned, sperm quality and function parameters were evaluated in both fresh and frozen–thawed samples in order to determine the effects of AQP inhibition during sperm cryopreservation. Regarding fresh samples, the absence of differences between controls and treated samples in any sperm parameter was due to the fact that inhibitors were added immediately before cryopreservation (*p* > 0.05; Table 1).

### 3.1. Sperm Motility

**Figure 1:**
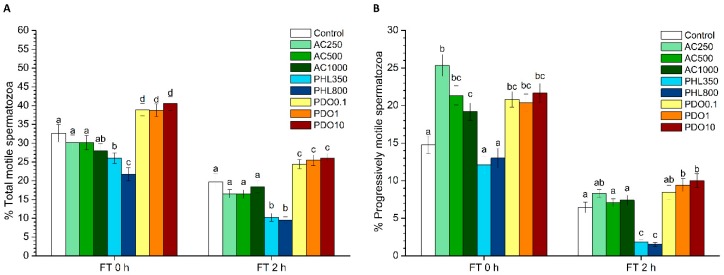
Sperm motility of samples after cryopreservation with a standard freezing medium (control) or with a freezing medium supplemented with acetazolamide (AC) at three different concentrations (250, 500, and 1000 µmol/L), with phloretin (PHL) at two different concentrations (350 and 800 µmol/L), or with 1,3-propanediol (PDO) at three concentrations (0.1, 1, and 10 mmol/L). (**A**) The percentages of total motile spermatozoa (TMOT). (**B**) The percentages of progressively motile spermatozoa (PMOT). Data, shown as mean ± SEM, correspond to 0 and 2 h post-thaw. Different letters (a–d) indicate significant differences (*p* < 0.05) between treatments within a given time point.

The total and progressive motilities of frozen–thawed spermatozoa are shown in Figure 1. The percentages of total motile spermatozoa were significantly (*p* < 0.05) higher in PDO treatments than in the control at both 0 and 2 h post-thaw (Figure 1A), whereas the two PHL concentrations caused a significant (*p* < 0.05) decrease in total motility at both post-thaw time points; the presence of AC did not cause any significant effect (*p* > 0.05).

Progressive sperm motility (Figure 1B) significantly (*p* < 0.05) increased in the presence of both AC and PDO immediately after thawing, whereas PHL did not have any significant effect (*p* > 0.05). Two hours after thawing, there were no significant differences between controls and samples treated with either AC or the lowest concentration of PDO (*p* > 0.05). In contrast, progressive sperm motility in the treatments containing 1 and 10 mmol/L of PDO was significantly (*p* < 0.05) higher than in the control. Moreover, the progressive sperm motility in the treatments containing PHL was significantly (*p* < 0.05) lower than in the control.

### 3.2. Sperm Viability: SYBR14/PI Test

**Figure 2:**
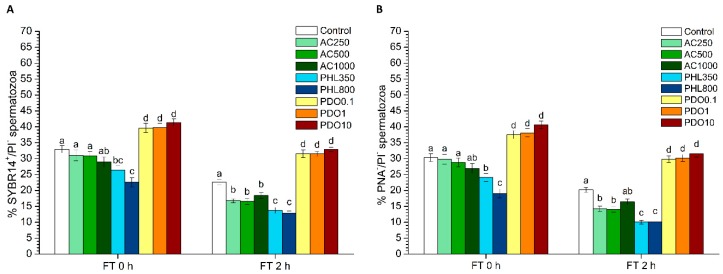
Sperm viability (SYBR14/PI) and acrosome integrity (PNA-FITC/PI) after cryopreservation with a standard freezing medium (control) or with a freezing medium supplemented with acetazolamide (AC) at three different concentrations (250, 500 and 1000 µmol/L), with phloretin (PHL) at two different concentrations (350 and 800 µmol/L), or with 1,3-propanediol (PDO) at three concentrations (0.1, 1 and 10 mmol/L). (**A**) The percentages of viable spermatozoa (SYBR-14^+^/PI^−^ spermatozoa). (**B**) The percentage of viable spermatozoa with an intact acrosome (PNA-FITC^−^/PI^−^ spermatozoa). Data, shown as mean ± SEM, correspond to 0 and 2 h post-thaw. Different letters (a–d) indicate significant differences (*p* < 0.05) between treatments within a given time point.

The percentages of viable spermatozoa (SYBR14^+^/PI^−^) did not differ between AC treatments and the control immediately after thawing (0 h), but the presence of this inhibitor induced a significant (*p* < 0.05) decrease at 2 h post-thaw (Figure 2A). While the percentages of viable spermatozoa in PHL treatments were significantly (*p* < 0.05) lower than in the control at 0 and 2 h post-thaw, all PDO treatments showed significantly (*p* < 0.05) higher percentages of viable spermatozoa than the control.

### 3.3. Acrosome Integrity: PNA-FITC/PI Test

The PNA-FITC/PI test was carried out to determine the integrity of both acrosome and plasma membranes, and PNA-FITC^−^/PI^−^ spermatozoa were those that had an intact acrosome and plasma membrane (Figure 2B). Samples treated with AC did not show significant differences to the control at any concentration immediately after thawing (*p* > 0.05), but there was a significant (*p* < 0.05) decrease in the percentage of spermatozoa that presented an intact plasma and acrosome membrane in the samples treated with the lowest concentrations of AC at 2 h post-thaw. The percentages of spermatozoa with an intact plasma and acrosome membrane were significantly (*p* < 0.05) lower in PHL treatments than in the control at both 0 and 2 h post-thaw. In contrast, all treatments containing PDO showed significantly (*p* < 0.05) higher percentages of spermatozoa with an intact plasma and acrosome membrane at both 0 and 2 h post-thaw.

### 3.4. Membrane Lipid Disorder: M540/YO-PRO-1 Test

**Figure 3:**
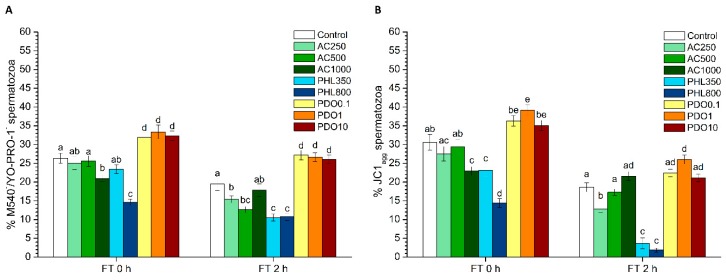
Sperm membrane lipid disorder (M540/YO-PRO-1) and mitochondrial membrane potential (JC1) after cryopreservation with a standard freezing medium (control) or with a freezing medium supplemented with acetazolamide (AC) at three different concentrations (250, 500 and 1000 µmol/L), with phloretin (PHL) at two different concentrations (350 and 800 µmol/L), or with 1,3-propanediol (PDO) at three concentrations (0.1, 1 and 10 mmol/L). (**A**) The percentages of viable spermatozoa with low membrane lipid disorder (M540^−^/YO-PRO-1^−^ spermatozoa). (**B**) The percentages of spermatozoa with high mitochondrial membrane potential (JC1_agg_ spermatozoa). Data, shown as mean ± SEM, correspond to 0 and 2 h post-thaw. Different letters (a–e) indicate significant differences (*p* < 0.05) between treatments within a given time point.

The M540/YO-PRO-1 test allowed for the evaluation of sperm membrane lipid disorder, the population of M540^−^/YO-PRO-1^−^ spermatozoa corresponding to those viable cells with low membrane lipid disorder (Figure 3A). While right after thawing, samples treated with 1000 µmol/L AC showed significantly (*p* < 0.05) lower percentages of viable spermatozoa with low membrane lipid disorder than the control, the treatments containing 250 and 500 µmol/L AC also showed reduced (*p* < 0.05) percentages of viable spermatozoa with low membrane lipid disorder than the control at 2 h post-thaw. The percentages of viable spermatozoa with low membrane lipid disorder at the highest PHL concentration were significantly (*p* < 0.05) lower than the control immediately after thawing. At 2 h post-thaw, both 350 and 800 µmol/L concentrations showed significantly (*p* < 0.05) lower percentages of viable spermatozoa with low membrane lipid disorder than the control. Finally, the percentages of viable spermatozoa with low membrane lipid disorder were significantly (*p* < 0.05) higher than the control in all PDO treatments, both at 0 and 2 h post-thaw.

### 3.5. Mitochondrial Membrane Potential (MMP): JC1 Test

The percentages of spermatozoa with high MMP were significantly (*p* < 0.05) lower in the treatment containing AC at 1000 µmol/L than in the control immediately after thawing and in the treatment containing AC at 250 µmol/L at 2 h post-thaw (Figure 3B). When treated with PHL, samples showed a decrease in MMP at any time point after thaw (*p* < 0.05). In contrast, the percentages of spermatozoa with high MMP in the treatment containing 1 mmol/L PDO were significantly (*p* < 0.05) higher than in the control both at 0 and 2 h post-thaw.

### 3.6. Intracellular Calcium of Spermatozoa: Fluo3/PI Test

**Figure 4:**
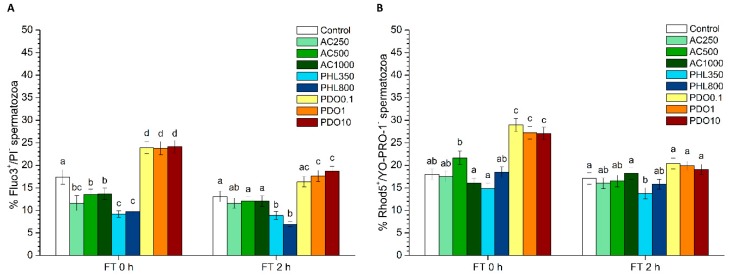
Intracellular calcium levels (Fluo3/PI and Rhod5/YO-PRO-1) after cryopreservation with a standard freezing medium (control) or with a freezing medium supplemented with acetazolamide (AC) at three different concentrations (250, 500 and 1000 µmol/L), with phloretin (PHL) at two different concentrations (350 and 800 µmol/L), or with 1,3-propanediol (PDO) at three concentrations (0.1, 1 and 10 mmol/L). (**A**) The percentages of viable spermatozoa with high levels of intracellular calcium levels (Fluo3^+^/PI^−^ spermatozoa). (**B**) The percentages of viable spermatozoa with high levels of intracellular calcium levels (Rhod5^+^/YO-PRO-1^−^ spermatozoa). Data, shown as mean ± SEM, correspond to 0 and 2 h post-thaw. Different letters (a–d) indicate significant differences (*p* < 0.05) between treatments within a given time point.

Intracellular calcium levels were assessed through the Fluo3/PI test, and the population of Fluo3^+^/PI^−^ spermatozoa corresponded to viable cells with high intracellular calcium levels (Figure 4A). While, compared to the control, samples treated with any concentration of AC showed a significant (*p* < 0.05) decrease in the percentage of viable spermatozoa with high levels of intracellular calcium immediately after thawing, no significant (*p* > 0.05) differences between AC treatments and the control were found after 2 h of thawing. Treatments containing any concentration of PHL showed significantly (*p* < 0.05) lower percentages of spermatozoa with high levels of intracellular calcium at both 0 and 2 h post-thaw. In contrast, the percentages of viable spermatozoa with high levels of intracellular calcium were significantly (*p* < 0.05) higher in all PDO concentrations than in the control right after thawing (0 h) and were significantly (*p* < 0.05) higher than the control at the two highest PDO concentrations after 2 h of thawing. 

### 3.7. Intracellular Calcium of Spermatozoa: Rhod5/YO-PRO-1 Test

The Rhod5/YO-PRO-1 test was also performed to determine intracellular calcium levels, and the population of Rhod5^+^/YO-PRO-1^−^ spermatozoa corresponded to viable cells with high intracellular calcium levels (Figure 4B). When AC was present, no significant (*p* > 0.05) differences with regard to the control were observed either at 0 or 2 h post-thaw. The treatments containing PHL showed no significant differences compared to the control at 0 h post-thaw, but those containing the lowest PHL concentration showed significantly (*p* > 0.05) lower percentages of viable spermatozoa with high intracellular calcium levels after 2 h of thawing. In contrast, the percentages of viable spermatozoa with high intracellular calcium levels were significantly (*p* < 0.05) higher in PDO treatments than in the control at 0 h but not at 2 h post-thaw. 

### 3.8. Intracellular Superoxide Levels (O_2_^−^): HE/YO-PRO-1 Test

**Figure 5:**
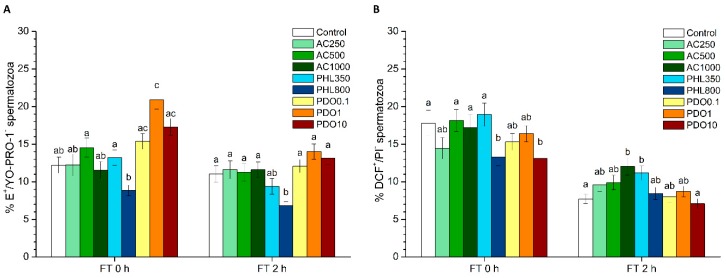
Intracellular levels of reactive oxygen species (ROS) after cryopreservation with a standard freezing medium (control) or with a freezing medium supplemented with acetazolamide (AC) at three different concentrations (250, 500 and 1000 µmol/L), with phloretin (PHL) at two different concentrations (350 and 800 µmol/L), or with 1,3-propanediol (PDO) at three concentrations (0.1, 1 and 10 mmol/L). (**A**) The percentages of viable spermatozoa with high levels of superoxide (E^+^/YO-PRO-1^−^ spermatozoa). (**B**) The percentages of viable spermatozoa with high levels of peroxide (DCF^+^/PI^−^ spermatozoa). Data, shown as mean ± SEM, correspond to 0 and 2 h post-thaw. Different letters (a–d) indicate significant differences (*p* < 0.05) between treatments within a given time point.

Intracellular O_2_^−^ levels were evaluated through the HE/YO-PRO-1 test, in which the population of E^+^/YO-PRO-1^−^ spermatozoa corresponded to viable cells with high intracellular levels of O_2_^−^. The percentages of viable spermatozoa with high intracellular levels of O_2_^−^ did not differ (*p* > 0.05) between AC treatments and the control, either at 0 or at 2 h post-thaw (Figure 5A). In contrast, the treatment containing 800 µmol/L PHL showed significantly (*p* < 0.05) lower percentages of viable spermatozoa with high intracellular levels of O_2_^−^ both at 0 and 2 h post-thaw (*p* < 0.05). Concerning samples treated with PDO, the single change that was observed was a significant (*p* < 0.05) increase in the percentages of viable spermatozoa with high intracellular levels of O_2_^−^ at 1 mmol/L immediately after thawing.

### 3.9. Intracellular Peroxide Levels (H_2_O_2_): H_2_DCFDA/PI Test

Intracellular H_2_O_2_ levels were assessed through the H_2_DCFDA/PI test, and the DCF^+^/PI^−^ sperm population corresponded to viable spermatozoa with high intracellular levels of peroxides. The percentages of viable spermatozoa with high intracellular levels of peroxides were significantly (*p* < 0.05) higher than the control in the treatment with 1000 µmol/L AC after 2 h of thawing (Figure 5B). While the percentages of viable spermatozoa with high intracellular levels of peroxides were significantly (*p* < 0.05) lower in the 800 µmol/L PHL treatment than in the control at 0 h post-thaw, those percentages in the treatment containing 350 µmol/L PHL were significantly (*p* < 0.05) higher than in the control after 2 h of thawing. Finally, the treatment containing 10 mmol/L PDO showed significantly (*p* < 0.05) lower percentages of viable spermatozoa with high intracellular levels of peroxides at 0 h post-thaw.

## 4. Discussion

While AQPs have been identified in sperm cells from different mammalian species in the last decade (reviewed in [2]), their precise function and mechanism of action are yet to be fully addressed. In the case of horses, AQP3, AQP7 and AQP11 have been identified in stallion spermatozoa [4]. Taking this into account, this study aimed to unveil the relevance of each group of AQPs during stallion sperm cryopreservation. With this purpose, three different inhibitors were added at different concentrations: AC, which is known to inhibit AQP1 and AQP4 [40,41]; PHL, which inhibits both AQP3 and AQP7 [42,43,44]; PDO, which has been proven to inhibit orthodox AQPs (AQP1, AQP2, AQP5 and AQP4) with high efficiency; and GLPs (the family to which AQP3, AQP7 and AQP9 belong) with low intensity [45,46]. The effects of each AQP-inhibitor on sperm function and survival after cryopreservation were assessed on the basis of sperm motility, sperm viability, acrosome integrity, membrane lipid disorder, MMP, intracellular calcium levels, and intracellular levels of ROS. Upon thawing, samples treated with AC showed higher PMOT and lower percentages of Fluo3^+^/PI^−^ than the control, and the highest AC concentration increased membrane lipid disorder and decreased MMP. At 2 h post-thaw, AC reduced sperm viability and the percentages of viable spermatozoa with low membrane lipid disorder at all concentrations. While the lowest AC concentrations reduced the percentage of acrosome-intact spermatozoa and MMP, the highest concentration of this inhibitor caused an increase in intracellular H_2_O_2_ levels. PHL decreased in all the studied parameters at almost any concentration and time point. In contrast, in the presence of PDO, samples showed higher percentages of TMOT, PMOT, viable and acrosome-intact spermatozoa, viable spermatozoa with low membrane lipid disorder, spermatozoa with high MMP, and viable spermatozoa with high intracellular calcium levels.

Differences between the effects of these three inhibitors appeared to result from their specificity for separate AQPs and from the collateral effects on other proteins that are present in the sperm cell. Considering the function of orthodox AQPs as water transporters and, as a consequence, their crucial role in osmoregulation, the expected effect of their inhibition through AC would be a drastic impairment of sperm quality and function. Since water outflow is crucial to avoid the formation of water crystals inside spermatozoa, the inhibition of water transport during cryopreservation could have dramatic consequences for the structure of sperm membranes and organelles, thereby compromising sperm membrane integrity and, thus, their function. In spite of this, there was an absence of clear effects immediately after thawing in the presence of AC, which is consistent with the fact that AQP1 and AQP4 have not been previously identified in stallion spermatozoa (reviewed in [2]). Nevertheless, AQP1 has been identified in boar spermatozoa [3]; therefore, while the few alterations in sperm function parameters that were observed in the presence of AC could be a consequence of this inhibition, future studies are necessary to assess whether this protein is present in stallion spermatozoa. Moreover, some of the changes observed in the presence of AC did not appear to depend on the concentration of the inhibitor or on the post-thaw incubation time. In this context, it is worth mentioning that not only does AC inhibit some AQPs but also carbonic anhydrase, whose function is to convert CO_2_ and H_2_O to bicarbonate and protons [47]. Therefore, the inhibition of carbonic anhydrase through AC could decrease the concentration of intracellular bicarbonate. Bicarbonate, together with Ca^2+^, is crucial for the activation of the soluble adenylate cyclase (sAC) which, in turn, produces cAMP that triggers protein kinase A (PKA). Protein kinase A activates different complexes of the electron transport chain, and the inhibition of PKA has been reported to reduce the electron flow passing through complex I [48]. This mechanism could explain the reduction of the mitochondrial membrane potential observed in the presence of some AC concentrations. In addition, the decrease in the percentages of spermatozoa with intracellular calcium levels observed in the presence of AC could be related to the lack of activation of the CatSper calcium channel. The CatSper channel is responsible for the increase of intracellular calcium levels that mediate sperm capacitation (reviewed in [49,50]), and a lower activation of this channel would also be related to the decreased levels of bicarbonate via the AC-inhibition of carbonic anhydrase. Since sperm motility is also mediated by the increase of intracellular bicarbonate and calcium levels, the higher sperm progressive motility observed right after thawing cannot be explained by these previous interactions. Thus, further research is needed to understand why sperm motility data observed in this work did not match with the decreased MMP and intracellular calcium levels observed immediately after thawing. Finally, while neither sperm viability nor acrosome integrity were altered at 0 h, both parameters decreased at 2 h post-thaw. This suggests that the aforementioned collateral effects of AC induce sub-lethal alterations that are not only apparent at 2 h post-thaw but not right after thawing.

The hydrophobic nature of PHL allows for its penetration through the sperm plasma membrane [51] and its binding to an internal site of GLPs [52]. This internal binding specifically inhibits GLPs, which in turn disrupts the transport of water and small solutes, such as glycerol. As glycerol, which is the most used permeating CPA in stallion sperm cryopreservation, was present in the freezing medium used in this study, it is reasonable to suggest that the addition of PHL decreased the transport of this CPA, which had detrimental effects on sperm quality and function parameters. The reduced influx of glycerol together with a limited water efflux through PHL-inhibition of GLPs could cause extreme osmotic stress to spermatozoa, thus compromising membrane integrity (including the membranes of intracellular organelles) and increasing membrane lipid disorder. Related with this, sperm motility and survival were lower than in the control after thawing, which could match with this hypothesis. In addition, an impairment in the sperm membrane integrity could also explain the drop in the intracellular calcium levels which, in turn, could be related to the compromised mitochondrial function that we observed through the decrease of spermatozoa with high MMP. While the reduction in the percentages of viable spermatozoa with high superoxide and peroxide levels immediately after thawing could be related to the decrease of MMP, the observed increase in the percentage of viable spermatozoa with high peroxide levels at 2 h post-thaw could be due to the inhibition of H_2_O_2_ efflux through AQP3 [53]. Related with this, some studies have unraveled that H_2_O_2_ efflux through AQP3 and AQP9 plays a vital role for human sperm function [11].

Regarding PDO, it strongly inhibits orthodox the AQPs remaining inside their pore [45,46]. PDO also inhibits GLPs, though less efficiently, since the pore diameter of GLPs is broader than that of orthodox AQPs. This broader diameter of GLPs allows PDO to pass through these channels [45,54]. Therefore, PDO would be able to impair the cell permeability to both water and small solutes, including glycerol, so that the expected effects should be a combination of those observed in the presence of AC and PHL. However, neither the cryodamage-related effects caused by the inhibition of GLPs that were observed in the presence of PHL nor the potential cryodamage that could occur as a consequence of the high-affinity inhibition of orthodox AQPs (if present in stallion spermatozoa) were observed in the presence of PDO. In fact, our results showed that the addition of PDO to the freezing medium had a positive effect on sperm quality and function parameters (motility, viability, acrosome integrity and membrane lipid disorder). On the one hand, the absence of a detrimental impact is consistent with the lack of negative effects observed in samples treated with AC; taking altogether, these results support—or, at least suggest—that orthodox AQPs are not relevant for stallion sperm cryotolerance. On the other hand, it is worth recalling that the affinity of PDO for GLPs is lower than for orthodox AQPs. Thus, its inhibitory efficiency might also be lower on orthodox AQPs, and, therefore, it might not be able to completely block water and solutes transport through the pore of this group of AQPs. Nevertheless, this absence of transport blockage does not explain the improving effect on the overall sperm quality and function in the presence of PDO. In this context, it could be hypothesized that PDO could function as a CPA itself, thereby mitigating the potential cryodamage that one would have expected to observe as a consequence of the lower intracellular concentration of glycerol. In fact, Widiasih et al. [55] observed that, when the impact of different cryoprotectants was evaluated, PDO yielded higher motility and viability in human spermatozoa than glycerol. Moreover, PDO has also been used as a CPA for the cryopreservation of canine ovarian cortex [56] and human multipotent stromal cells [57]. Considering all the aforementioned information, one could consider that the combination of two different CPAs at low concentrations could restrict the widely known collateral toxic effects of each CPA, including glycerol (reviewed in [50]). In fact, a less toxic and more efficient combination of CPAs would limit cryodamage and thus membrane alterations, which would yield higher post-thaw sperm viability and motility. The rise in the percentages of viable spermatozoa with high intracellular calcium levels could be related to a better maintenance of sperm survival rather than to the specific effect of PDO on the calcium transport (reviewed in [49,50]). In this context, while the increase in both total and progressive motilities and in MMP could be due to this calcium increase, the cause of this augmented calcium levels remains unexplained, since sperm membranes were intact according to the results of separate tests (SYBR14/PI, PNA-FITC/PI and M540/YO-PRO-1). Finally, the increase in superoxide levels might be a direct consequence of the increased MMP, since mitochondria are the main source of ROS. Considering all the aforementioned information, it can be hypothesized that PDO is not an effective inhibitor of GLPs and that the absence of negative effects is consistent with the results observed in the presence of AC. This suggests that orthodox AQPs might not be present in stallion spermatozoa or, if present, this group of AQPs is not relevant for stallion sperm cryotolerance. Moreover, further studies are needed to elucidate whether the use of PDO as a CPA alone or in combination with other agents might lead to obtaining frozen–thawed stallion spermatozoa of better quality.

## 5. Conclusions

In conclusion, the effects of AQP inhibition rely on the specificity of each inhibitor for each AQPs group and its collateral effects on other sperm proteins. The observed effects when samples were supplemented with AC, which mainly inhibits orthodox AQPs, suggest that these proteins are not involved in the response to osmolality changes produced during stallion sperm cryopreservation, and the observed changes seem to be caused by the side-effects on other sperm proteins. The absence of orthodox AQPs in stallion spermatozoa is consistent with the absence of detrimental effects in the presence of PDO, which is also a high-affinity inhibitor of orthodox AQPs. On the other hand, the dramatic impairment of post-thaw sperm quality observed in the presence of PHL suggests that GLPs play a crucial role in the response of stallion sperm to the osmolality changes that occur during cryopreservation. Finally, the improvement of overall sperm quality and function parameters in the presence of PDO evidences it might not be an efficient GLPs inhibitor and supports its role as a permeable CPA, whether alone or in combination with other cryoprotectants.

## Figures and Tables

**Table 1:** Sperm quality and function parameters (mean ± SEM) in fresh stallion semen. SYBR14^+^/propidium iodide (PI^−^) spermatozoa, viable cells; peanut agglutinin conjugated with fluorescein isothiocyanate (PNA-FITC^−^)/PI^−^ spermatozoa, cells with an intact plasma membrane and acrosome; merocyanine 540 (M540^−^)/YO-PRO-1^−^ spermatozoa, live cells with low mitochondrial membrane disorder; JC1_agg_ spermatozoa, cells with high mitochondrial membrane potential (MMP); Fluo3^+^/PI^−^ spermatozoa, live cells with high intracellular calcium levels, mainly in the sperm mid-piece; Rhod5^+^/YO-PRO-1^−^ spermatozoa, live cells with high intracellular calcium levels, mainly in the sperm head; E^+^/YO-PRO-1^−^ spermatozoa, live cells with high superoxide (O_2_^−^) levels; 2’,7’-dichlorofluorescein (DCF^+^)/PI^−^ spermatozoa, live cells with high peroxide (H_2_O_2_) levels.

Sperm Parameter	Mean ± SEM
% Total motile spermatozoa (TMOT)	78.4 ± 3.6
% Progressively motile spermatozoa (PMOT)	57.9 ± 2.4
% SYBR14^+^/PI^−^ spermatozoa	80.5 ± 3.2
% PNA-FITC^−^/PI^−^ spermatozoa	79.1 ± 3.0
% M540^−^/YO-PRO-1^−^ spermatozoa	76.2 ± 3.2
% JC1_agg_ spermatozoa	73.6 ± 3.1
% Fluo3^+^/PI^−^ spermatozoa	14.3 ± 0.9
% Rhod5^+^/YO-PRO-1^−^ spermatozoa	20.8 ± 1.4
% E^+^/YO-PRO-1^−^ spermatozoa	8.7 ± 0.7
% DCF^+^/PI^−^ spermatozoa	4.5 ± 0.4
